# Soil Microbial Community, Soil Quality, and Productivity along a Chronosequence of *Larix principis-rupprechtii* Forests

**DOI:** 10.3390/plants12162913

**Published:** 2023-08-10

**Authors:** Jing Zhang, Qiang Liu, Dongzhi Wang, Zhidong Zhang

**Affiliations:** College of Forestry, Hebei Agricultural University, Baoding 071001, China; zj_0882@163.com (J.Z.); qiangliu2015@126.com (Q.L.); wangdz@126.com (D.W.)

**Keywords:** stand age, soil nutrients, soil microbial community, *Larix principis-rupprechtii*, productivity

## Abstract

Elucidating the correlation between soil microbial communities and forest productivity is the focus of research in the field of forest ecology. Nonetheless, the relationship between stand age, soil quality, soil microorganisms, and their combined influence on productivity is still unclear. In this study, five development stages (14, 25, 31, 39, and >80 years) of larch (*Larix principis-rupprechtii*) forests were investigated in Inner Mongolia and Shanxi provinces of China. We evaluated soil quality using the Integrated Soil Quality Index (SQI) and analyzed changes in bacterial and fungal communities using high-throughput sequencing. Regression models were also established to examine the impacts of stand age, microbial diversity, and SQI on productivity. The findings revealed an ascending trend in soil organic matter (SOM), total nitrogen (TN), total phosphorus (TP), available potassium (AK), and SQI in 14, 25, 31, and 39-year-old stands. The abundance of oligotrophic bacteria Acidobacteria exhibited a gradual decline with increasing forest age, whereas copiotroph bacteria Proteobacteria displayed a progressive increase. Stands older than 80 years exhibited a higher abundance of both the saprophytic fungus Ascomycota and mycorrhizal fungus Basidiomycota. Forest age had a significant impact on microbial diversity, particularly in terms of bacterial diversity, impacting both α and β diversity. The soil bacterial community structure was influenced by AK, SOM, TN, TP, and pH. Conversely, the fungal community structure was regulated by crucial factors including SOM, TN, TP, TK, AK, and pH. Fungal diversity demonstrated a significant and positive correlation with the basal area increment (BAI) of larch. Furthermore, microbial diversity accounted for 23.6% of the variation in BAI. In summary, the findings implied a robust association between microbial composition, diversity, and soil chemical properties throughout the chronosequence of larch forests. These factors collectively played a crucial role in influencing the productivity of larch forest.

## 1. Introduction

Afforestation plays an important role in carbon sequestration. However, the continuous planting of single-species forests or extensive tree harvesting in the same area can lead to soil quality degradation and reduced forest productivity [1], a phenomenon known as the continuous pure forest planting problem (CMP) [2,3]. Extensive research has been conducted on CMP in Chinese plantations, and some studies have suggested that the decline in forest productivity is associated with shifts in soil nutrient cycling, such as the transition from nitrogen limitation to phosphorus limitation [4]. Therefore, with increasing emphasis on sustainable forestry management, it is important to assess the impact of soil quality.

Soil quality plays a vital role in plantations as it directly influences biomass production and carbon sequestration [5,6]. However, previous studies have primarily focused on individual soil physicochemical properties rather than evaluating soil quality comprehensively [7,8]. The soil quality index (SQI) has emerged as a widely used approach to assess integrated soil quality by considering multiple indicators and assigning them appropriate weights [9]. The selection of these indicators varies across different studies and contexts. For instance, in a study on fir plantations, Chen et al. [1] identified total nitrogen (TN), acid phosphatase (ACP), dissolved organic nitrogen (DON), and total potassium (TK) as key indicators of soil quality in the topsoil. Although some studies have examined the effects of forest age on soil physicochemical properties and microbial community composition, a comprehensive assessment of soil quality throughout the life cycle of larch (*Larix principis-rupprechtii* Mayr) plantations is currently lacking.

Microorganisms play pivotal roles as both performers and drivers of material cycles and energy flows in soil ecosystems. They play a vital role in promoting organic degradation, nutrient mineralization, plant nutrient acquisition, and growth promotion [10,11]. By acquiring resources and influencing the flow of ecosystem materials, microorganisms actively regulate and maintain ecosystem functions [12]. Conversely, plants exert a strong selective effect on soil microbial communities through specific apoplastic chemical reactions [13]. Numerous studies have demonstrated the influence of soil physicochemical properties on microbial structure and composition. For example, Liu et al. [14] revealed that 25 years of acacia afforestation resulted in the rapid formation of a distinct understory microhabitat, altering microbial community composition through phosphorus availability. Van Der Heijden et al. [15] suggested that reduced nutrient availability in the soil may promote fungal rather than bacterial growth due to the competitive advantage of fungi in the uptake of nitrogen and phosphorus. Wang et al. [16] concluded that soil physicochemical properties, particularly pH, DON, and TN, played critical roles in shaping the composition and diversity of soil fungal and bacterial communities along forest age gradients in subtropical China. Additionally, microbial communities are essential regulators of biogeochemical cycles. Understanding their contributions to ecosystem processes is vital for predicting forest responses to future environmental conditions [17,18].

Soil microorganisms play a crucial role in regulating forest productivity, particularly in nutrient-poor ecosystems where plant symbionts are essential for acquiring limited nutrients [15]. It has been demonstrated that mycorrhizal fungi and nitrogen-fixing bacteria can provide up to 90% of the required phosphorus and nitrogen for plants [19,20]. However, soil microorganisms can also have negative impacts on forest productivity by acting as pathogens, competing with plants for nutrients, or converting nutrients into forms that are inaccessible to plants. Interactions between plants and microbes play a central role in soil fertility, biogeochemical cycling, and related ecosystem functions, yet the exact mechanisms by which microorganisms influence forest productivity remain a topic of debate. Studies have shown that fungal diversity positively correlates with forest productivity, diversity, and nutrient acquisition [19], but some studies have also suggested that productivity increases are determined by species attributes rather than overall diversity [21]. The majority of previous studies have focused on fungal diversity, while exactly how bacterial diversity affects forest productivity remains unclear.

Larch is a prominent afforestation species in northern China, with the advantages of fast growth, superior wood quality, soil and wind retention, and high resilience to climate change. However, it faces significant challenges, including low forest quality, limited productivity, and instability, which hinder the effective utilization of the multifunctional benefits offered by forests [22]. Most previous studies have focused on how aboveground biomes influence productivity, while often neglecting the role of belowground soil biomes, which are intricately linked to plant growth and nutrient utilization [23].

To uncover the primary factors influencing the productivity of larch forests in northern China, we conducted soil samples across different stand age gradients (14, 25, 31, 39, and >80 years), allowing us to examine soil chemical properties, microbial composition, and diversity at various stages of forest development. The purpose of this study was to answer the following three questions: (1) how does stand age impact SQI, microbial community composition, and diversity; (2) whether soil chemical properties have distinct effects on the composition and diversity of fungal and bacterial communities; and (3) what are the coupled effects of stand age, SQI, and microbial community diversity on productivity. The objective of this study is to provide a scientific foundation for the sustainable management of larch forests.

## 2. Results

### 2.1. Changes in BAI with Stand Age

**Figure 1:**
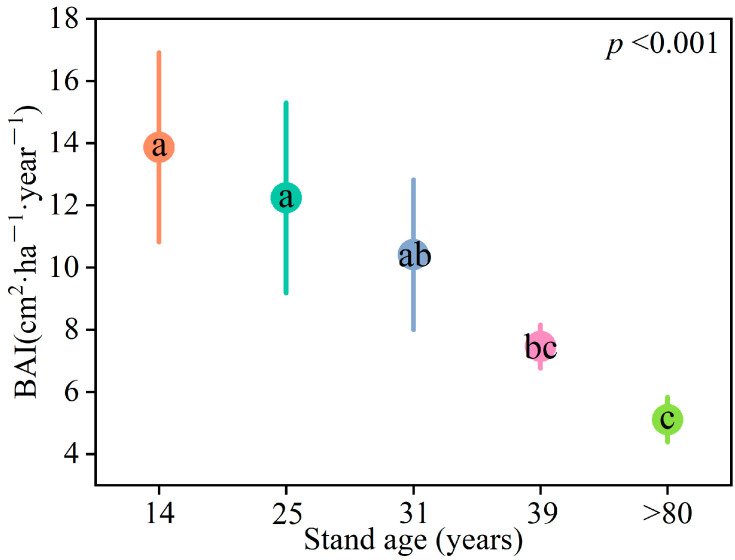
Difference in BAI of larch stands with different ages. Different letters indicate significant differences among stand ages. Different colors represent different stand ages.

The BAI (basal area increment) of larch stands exhibited a gradual decline with increasing stand age. Specifically, the 14 and 25-year-old stands demonstrated significantly higher BAI compared to the 39 and >80-year-old stands (*p* < 0.05) (Figure 1).

### 2.2. Changes in Soil Chemical Properties and SQI with Stand Age

Table 1 shows the significant increases in soil SOM, TN, TP, and AK from 14 to 39-year-old stands, with average increases of 78%, 66%, 66%, and 41% compared to the 14-year-old stand, respectively. The effect of stand age on TK was not found to be significant. The content of AP was relatively high in the 31 and 39-year-old stands. It is worth noting that the soils in all stands of different ages were found to be acidic.

**Figure 2:**
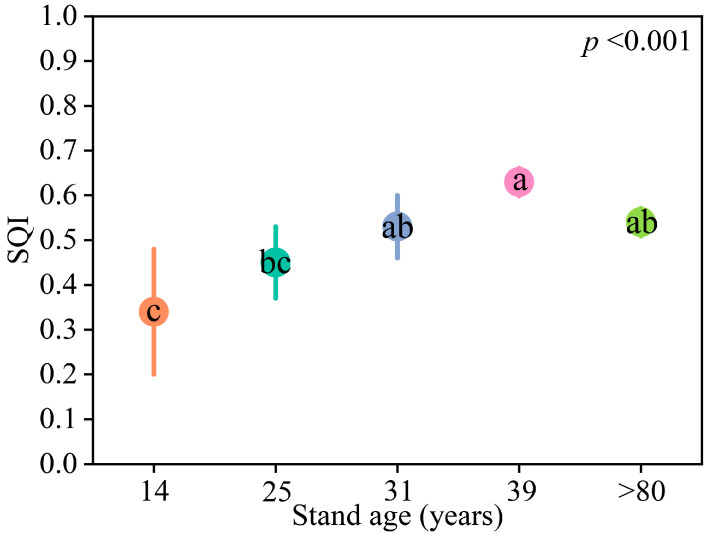
Difference in SQI of larch stands with different ages. Different letters indicate significant differences among stand ages. Different colors represent different stand ages.

A principal components analysis (PCA) revealed that the first two principal components accounted for 75.22% of the variation in soil nutrients, and all of the principal components had eigenvalues ≥ 1.0 (Table S1). The indicators SOM, TN, and TP were highly weighted in PC1 and were significantly correlated with one another. TN had the highest weight (0.96) of these indicators and thus was included in the minimal data set (MDS). TK had the highest weight (0.92) in PC2 and was also retained in MDS (Table S2). The SQI was calculated based on the weighted values of PCA. The SQI increased gradually with stand age from 0.34 at 14 years to 0.63 at 39 years. The SQI decreased slightly in stands older than 80 years but was not significantly different from the 39-year-old stands (Figure 2).

### 2.3. Changes in Microbial Community Composition and Diversity with Stand Age

**Figure 3:**
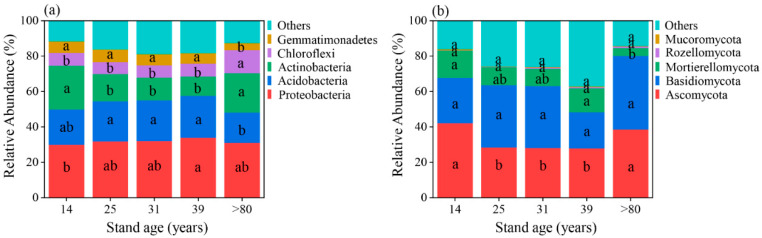
Relative abundance (%) of the most dominant bacterial (**a**) and fungal (**b**) groups at the phyla level in larch chronosequence. Different letters indicate significant differences among stand ages.

At different stages of stand development, Proteobacteria (31.75%), Acidobacteria (21.03%), Actinobacteria (17.53%), Chloroflexi (8.32%), and Gemmatimonadetes (5.88%) were found to be the dominant phyla of bacteria in the stand. (Figure 3a, Table S3). The effect of stand age on all five dominant bacterial phyla was found to be significant (*p* < 0.05). In terms of fungal composition, the dominant phyla in all development stages were Ascomycota (33.05%), Basidiomycota (31.58%), and Mortierellomycota (10.7%) (Figure 3b, Table S3). The effect of stand age on Ascomycota and Mortierellomycota was found to be significant (*p* < 0.05).

**Figure 4:**
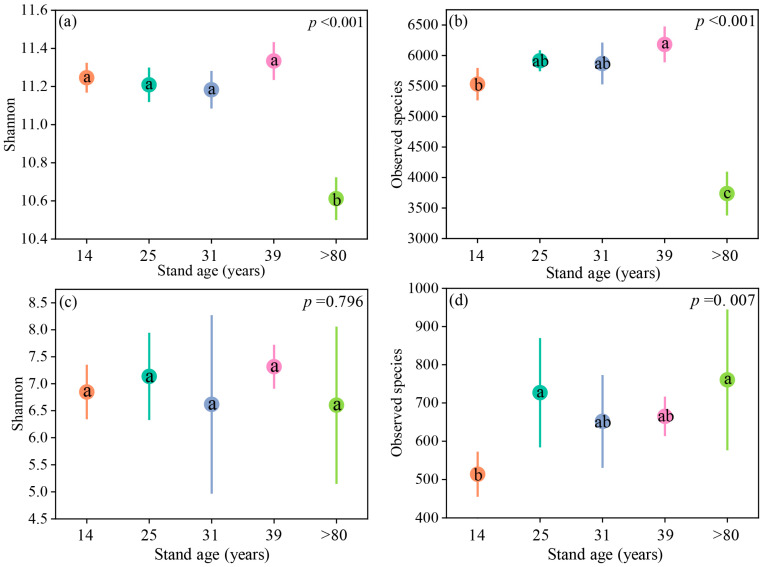
Differences in α-diversity of bacteria (**a**,**b**) and fungi (**c**,**d**) among different ages in larch forests. Different letters indicate significant differences among stand ages. Different colors represent different stand ages.

The bacterial α-diversity indices, including the richness index (observed species) and Shannon index, showed significant differences among the different forest ages. However, only the observed species index exhibited significant differences among the fungi. Specifically, both the Shannon index and observed species index were significantly higher at 14 years compared to 80 years (*p* < 0.001) in bacterial communities, (Figure 4a,b). As for fungi, there was no significant difference in the Shannon index across different stand ages (Figure 4c), while the observed species index was significantly lower at 14 years compared to both 25 years and >80 years (Figure 4d).

**Figure 5:**
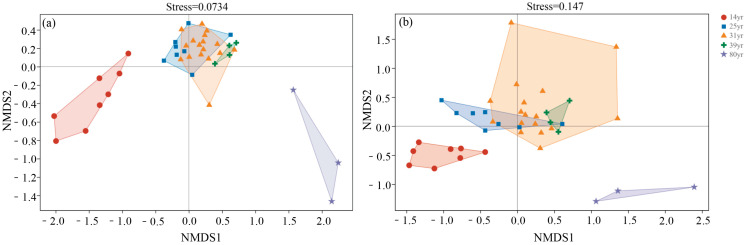
β-diversity of bacterial (**a**) and fungal (**b**) in larch stands with different ages. Non-metric multidimensional scaling (NMDS) analysis based on ASV data sets was used to calculate β-diversity using the Bray–Curtis distance matrix.

With respect to β-diversity, the bacterial communities showed significant differences (*p* < 0.05) between the different stand ages (Figure 5a). In contrast, fungal β-diversity was not significant in stands between 31 and 39 years of age, but did show significant differences (*p* < 0.05) between other stand ages (Figure 5b).

### 2.4. Relationship between Microbial Communities and Soil Chemical Properties

**Figure 6:**
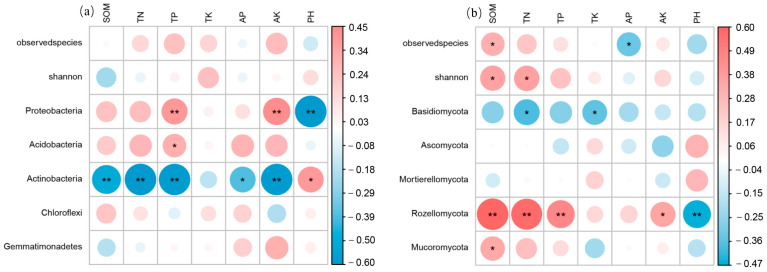
Spearman correlation coefficients between alpha diversity and the dominant species (relative abundance) of bacterial (**a**) and fungal (**b**), and soil characteristics. The colors represent positive and negative correlations (red: positive; blue: negative), and the numbers represent correlation coefficients. ** (*p* < 0.01) and * (*p* < 0.05).

Alpha diversity of the bacterial community was not significantly correlated with soil properties (Figure 5a). Alpha diversity of the fungal community, however, was significantly positively correlated with SOM (soil organic matter) (*p* < 0.05). Furthermore, the observed fungal species index was significantly negatively correlated with AP (available phosphorus) (*p* < 0.05), while the Shannon index was found to be significantly positively correlated with TN (total nitrogen) (*p* < 0.05) (Figure 6b).

**Figure 7:**
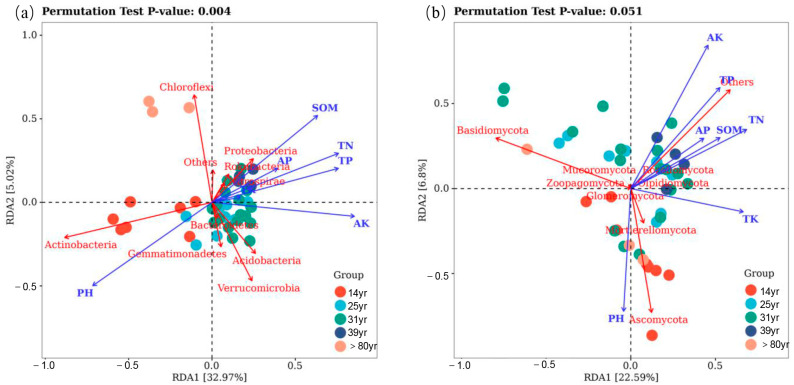
Redundancy analysis (RDA) shows the effects of soil factors on bacterial (**a**) and fungal (**b**) phyla in the larch chronosequence. The ordination is based on Bray–Curtis distance with forward selection, and factors were chosen that significantly (*p* < 0.05) contributed to the model.

The RDA results indicated that the age of the stand and chemical properties of the soil explained 37.08% of the total variation in bacterial community composition at the phylum level (Figure 7a), as well as 28.78% of the total variation in fungal community composition at the phylum level (Figure 7b). Among the soil chemical properties, AK (available potassium), SOM, TN, TP (total phosphorus), and pH were identified as significant factors influencing the composition of bacterial phylum communities. Similarly, SOM, TN, TP, TK (total potassium), AK, and pH were significant factors affecting the composition of fungal phylum communities (Table S4).

### 2.5. The Interaction between Stand Age, Soil Microbial Diversity, and Soil Quality Has an Impact on BAI

**Figure 8:**
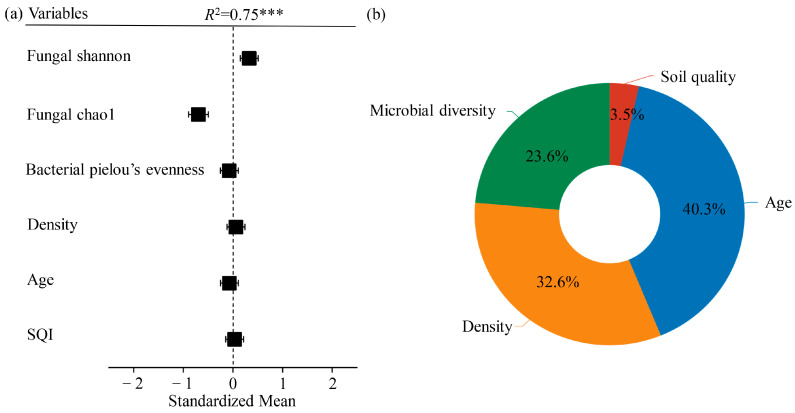
(**a**) Variance contribution rate: fungal Shannon, fungal chao1, bacterial Pielou’s evenness, density, age, and SQI to BAI of larch *** (*p* < 0.001); and (**b**) the relative contributions of density, age, microbial diversity, and soil quality.

The multiple regression model revealed significant positive correlations between BAI (basal area increment) and age, fungal Shannon index, density, and SQI. Conversely, fungal Chao1, bacterial Pielou’s evenness, and age exhibited significant negative correlations with BAI (*p* < 0.05, Figure 8a, Table S5). The factors contributing to the variance in BAI, ranked by percentage contribution in decreasing order, were age (40.3%), density (30%), microbial diversity (23.6%), and soil quality (3.5%) (Figure 8b).

## 3. Discussion

### 3.1. Changes in Soil Chemical Properties with Stand Age

The chemical properties of the soil show significant variation with forest age, which is consistent with previous studies [16]. Forests play a vital role as carbon sinks, and human activities such as deforestation, forest management, and afforestation have greatly influenced forest carbon balance over thousands of years [24]. Our findings suggest that soil nutrient content is lower in 14-year-old stands (Table 1), which could be attributed to factors like soil erosion or organic matter decomposition resulting from previous deforestation [25], or a decrease in understory vegetation and litter nutrient return in younger stands [16]. Guillaume et al. [26] demonstrated that the conversion of tropical rainforests into plantations resulted in a loss of 10 mg·C·ha^−1^ after about 15 years. The increase in SOM observed with increasing stand age (14–39 years) (Table 1) may be attributed to the humification of plant residues, as well as the input of carbon through litter and root deposition by photosynthesis [27]. The increase in TN in soil is mainly due to the decomposition of surface litter and the deposition of atmospheric nitrogen [28]. There is a strong relationship between organic carbon and nitrogen. The maintenance of long-term carbon pools requires continuous input of nitrogen, as nitrogen deficiency can lead to increased carbon mineralization [29]. Plants acquire mineral phosphorus and potassium from deeper soil layers [30], transporting them from roots to shoots and subsequently depositing organic phosphorus and potassium as litter in the topsoil [31]. Soil AP content decreases in 14 and 25-year-old stands, probably due to the rapid growth of younger trees requiring soil AP uptake. As the stand develops and litter accumulates, the soil AP content tends to increase. A previous study indicated that phosphorus uptake in fir plantations increased by 2.2–2.8 kg·ha^−1^ in one year [30]. Overall, soil nutrient content gradually increases with increasing stand age, but decreased gradually from mature to old-growth stands, possibly due to the higher nutrient demand, particularly for nitrogen and phosphorus, in old-growth stands [32].

### 3.2. Changes in Soil Microbial Community with Stand Age

The microbial composition of larch plantations varied across different stand ages. Acidobacteria, Proteobacteria, and Actinobacteria were the dominant bacterial communities in the soil (Figure 3a), whereas soil fungal communities were dominated by Ascomycota and Basidiomycota across the range of age gradients (Figure 3b). These findings were confirmed in previous studies [16,33]. Changes in microbial community composition reflect shifts in soil ecology and function [34]. Acidobacteria are generally recognized as oligotrophic bacteria, and their relative abundance tends to increase with decreasing soil nutrient content [25]. This pattern may correspond to the k-strategy ecological strategy of Acidobacteria. Proteobacteria are considered copiotrophic bacteria and are typically more abundant in nutrient-rich soils (33.92% at 39-year-old stand). However, our study revealed that the abundance of Acidobacteria was low in 14 and >80-year-old stands, while the abundance was high in 25, 31, and 39-year-old stands (Figure 3a), following a similar trend as TN content. These results differ from previous findings [16], which may be attributed to the overall high soil nutrient content in our study sites, resulting in non-significant changes in the relative abundance of Acidobacteria across different stand ages. Of note, the relative abundances of Proteobacteria were consistently higher than Acidobacteria, lending further support to this interpretation. The relative abundance of Proteobacteria is usually closely related to soil carbon content [35], and the higher the soil carbon content, the higher the relative abundance of Proteobacteria, which is consistent with previous findings [36]. While Actinobacteria are generally considered to be both copiotrophic and oligotrophic organisms [37], the results in the present study lean more towards an oligotrophic lifestyle. The reasons for this discrepancy are unclear and require further investigation.

Ascomycota fungi are known as free-living saprophytes that play a role in the decomposition of litter and organic matter. They are often characterized as cellulolytic or sugar fungi with limited capacity for lignin decomposition [38]. In our study, the relative abundance of Ascomycota was higher in stands older than 80 years and lower in 25, 31, and 39-year-old stands (Figure 3b). This pattern may be attributed to the accumulation of substantial litter material in stands older than 80 years. On the other hand, Basidiomycota exhibited the highest relative abundance in >80-year-old stands (Figure 3b). Basidiomycota fungi are mycorrhizal and have a greater inclination for lignin decomposition, which is more challenging to break down in litter matter [39]. This ability promotes nutrient uptake by plants. Generally, the relative abundance of Basidiomycota is higher in low-nutrient soils. The accumulation of non-degradable organic carbon may be associated with the needle-like leaf shape and high lignin content of larch [40].

Prior studies have demonstrated that changes in soil microbial communities are influenced by a variety of factors including soil physicochemical properties [41], litter biomass, vegetation cover, and fine root biomass [25]. Our results showed that bacterial α-diversity gradually increased with increasing stand age from 14 to 39 years (Figure 4a). This pattern could be attributed to an increase in soil nutrients resulting from litter input, indirectly influencing the diversity of soil bacterial communities [42,43], as has been previously reported. In comparison to medium-aged stands (12, 18, and 25 years), low (3 and 6 years), and high-aged stands (32 and 49 years) exhibited higher nutrient content, enzyme activity, microbial richness, and diversity [36]. Bacterial α-diversity showed a decreasing trend at >80 years (Figure 4a), possibly due to competition between tree growth and microorganisms stemming from reduced soil nutrients. The significant change in bacterial richness compared to diversity with increasing stand age is similar to the findings of Liu’s study [14]. This pattern could be attributed to interspecific competition among bacteria, which is influenced by soil nutrient availability. Our study showed no significant change in fungal diversity with increasing stand age (Figure 4c,d), which may be due to the greater adaptability of fungi to their environment compared to bacteria. For example, many fungal species can produce spores that efficiently utilize nutrients during their germination phase, especially in nutrient-poor environments [44].

### 3.3. Relationship between Soil Quality and Microbial Community

Soil chemical properties and environmental factors are known to be key drivers of microbial communities in forest ecosystems [45]. Microorganisms play a crucial role in ecological processes and can serve as indicators of soil quality [46]. The results of the RDA analysis and correlation thermogram revealed that soil properties such as AK, SOM, TN, TP, and pH were the main factors influencing the composition of soil bacterial communities at different forest ages (Figure 7a). In particular, pH significantly affected the relative abundance of the dominant bacterial phyla, Proteobacteria, and Actinobacteria (Figure 6a), which is consistent with findings by Zhao et al. [47]. The wide pH range observed among the sampling sites was found to be a prerequisite for pH-driven changes in the bacterial community. The significant positive correlation (*p* < 0.01) between Proteobacteria and TP and AK may be attributed to their symbiotic lifestyle (r-strategy) [48].

Regarding soil fungal communities, soil properties such as TK, AK, SOM, TN, TP, and pH were identified as the main factors influencing their composition at different stand ages (Figure 6b and Figure 7b). Notably, SOM, TN, TP, and Rozellomycota showed significant positive correlations (*p* < 0.01), highlighting the role of fungi in releasing soil carbon, nitrogen, and phosphorus for subsequent tree uptake. Fungi generally thrive in acidic environments, and the lower pH of the soil increases the solubility of organic matter and changes the composition of dissolved organic matter, thereby influencing fungal community structure within the soil [49]. For example, Grantina et al. [50] found that the abundance and richness of fungi in acidic soils in forests (pH 4.46~5.30) was significantly greater than that in arable soils (pH 6.96~7.69), indicating that a lower pH promotes soil fungal growth in general. It is important to note that soil pH itself does not directly drive microbial community changes but rather interacts with other soil variables such as enzyme activity, nutrient availability, organic carbon properties, salinity, or soil moisture.

In summary, the shifts in the composition of soil bacterial and fungal communities are closely linked to changes in soil quality, highlighting the intricate relationships between microbial communities and soil properties.

### 3.4. Effects of Stand Structure, Soil Microbial Diversity, and Soil Quality on Productivity of Larch

The multiple regression analysis conducted in this study revealed a significant and positive correlation between fungal diversity and BAI (basal area increment) (*p* < 0.05) (Figure 8, Table S5). It was found that soil microbial diversity played an equally important role as stand structure (stand age and stand density) in determining productivity. This finding is consistent with previous research indicating that fungal diversity is associated with increased plant productivity, plant diversity, and nutrient acquisition [51]. The positive relationship between fungal diversity and productivity can be attributed to the influence of microorganisms on the decomposition of litter material in larch, which releases essential nutrients required for tree growth. Microbial diversity can facilitate decomposition through various mechanisms. Saprophytic fungi primarily decompose fresh surface litter material, contributing to carbon mineralization. Conversely, mycorrhizal fungi dominate the lower soil layers and are more inclined to decompose already decayed litter material and humus, thereby mobilizing nitrogen and transporting it to host plants [36].

SQI serves as a comprehensive indicator reflecting various soil properties, including soil texture, organic matter content, pH, effective nutrient content, and microbial activity. These properties have significant effects on soil fertility, permeability, water retention, and disease resistance [38]. Consequently, a higher SQI generally indicates a healthier, more fertile, and more favorable soil environment for plant growth and development. The observed increase in forest productivity of larch can be attributed to the fact that soils with high SQI contain more nutrients and water, thereby providing optimal growing conditions. Moreover, soils with high SQI can support diverse microbial communities that form symbiotic relationships with plants and promote plant growth and nutrient uptake [1]. Enhancing SQI can be achieved through appropriate land management measures such as fertilization, improving soil structure, and increasing organic matter content, which in turn contribute to improved forest productivity and overall ecosystem health.

## 4. Materials and Methods

### 4.1. Study Area

**Figure 9:**
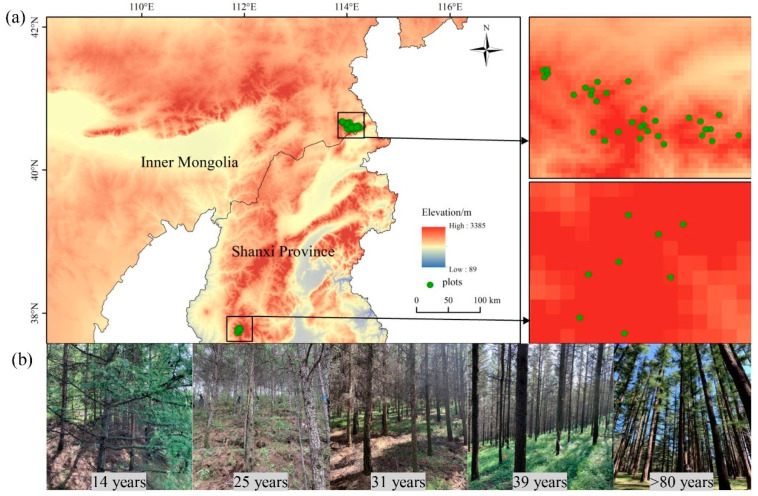
(**a**) The location of study sites of larch plantations in Inner Mongolia and Shanxi Province in northern China and (**b**) pictures of five age stands.

The study area is located in the Sumushan Scenic Area in Inner Mongolia Autonomous Region (40°26′–40°39′ N, 113°38′–114°02′ E), and Pangquangou National Nature Reserve in Shanxi Province (37°45′–37°55′ N, 111°22′–111°33′ E) (Figure 9). The Sumushan Scenic Area has a continental monsoonal temperate climate. Mean annual temperatures range from 0 to 6 °C, and rainfall ranges from 150 to 450 mm, respectively. The dominant tree species in this area include larch, pine (*Pinus sylvestris* L.), spruce (*Picea asperata* Mast.), birch (*Betula platyphylla* Suk.), and aspen (*Populus* L.). The Pangquangou National Nature Reserve has a mean annual temperature of 5 °C, with a mean growing season temperature of 16.5 °C, and mean annual rainfall of 822.6 mm. Its climate is characterized as a continental mountain monsoon climate. The primary forest types in this region are coniferous forest, broadleaf forest, and mixed conifer forest. Major tree species include larch, birch, spruce, and red birch (*Betula albosinensis* Burk.).

### 4.2. Site Survey and Sampling

A total of 40 sampling plots of pure larch stands in five development stages (14, 25, 31, 39, and >80 years) were surveyed in 2019. The samples we took in Shanxi were all >80 years old, while the remaining samples were taken in Inner Mongolia. The sampling plots were 30 m× 30 m in size, and latitude, longitude, and altitude of each plot were recorded. We identified and numbered all standing trees that had a diameter at breast height (DBH) ≥5 cm in height. Tree species names, DBHs, and heights were measured and recorded. See Table 2 for details.

The number of tree cores obtained from each sampling plot was determined based on the diameter distribution. At breast height (1.3 m), two orthogonal tree cores in the eastern and northern directions were carefully extracted using an increment border. The cores of the trees were placed in plastic tubes and sealed with paper tape to be stored temporarily, and 480 drilled core samples were taken to the laboratory for further processing. In the laboratory, the cores were fixed and subjected to preliminary cross-dating. Each sample was then scanned, and the width of the annual rings was measured using WinDENDRO software. Cross-dating tests and quality control were conducted using the COFECHA program [52].

In each sampling plot, soil samples were obtained by removing the surface litter. A five-point sampling method was employed, and a soil auger with an internal diameter of 4 cm was used to extract soil from the top 0–10 cm depth. The extracted soil was then combined to create one composite soil sample per plot. A total of 80 soil samples were collected, with two samples taken from each of the 40 sample plots. One portion of the soil samples was processed by filtering out plant roots and stones using a sieve. These filtered samples were then packed into 20 mL centrifuge tubes and stored in a −80 °C refrigerator for subsequent high-throughput sequencing analysis. The other portion of the samples was spread out on a cool laboratory bench and left to naturally air dry. These dried samples were utilized for determining soil chemical properties.

### 4.3. Analysis of Soil Chemical Properties

Soil organic matter (SOM) determination: A 0.4 mol/L potassium dichromate solution was prepared and placed in an oil bath at 180 °C. The soil samples were boiled in the solution for 5 min, and to determine the amount of potassium dichromate consumed, which represents the organic matter content, the remainder of the potassium dichromate was titrated with a solution of 0.2 mol/L of ferrous sulfate. Determination of total nitrogen (TN): About 1.000 g of air-dried soil sample was collected and mixed with 2 g of catalyst (K_2_SO_4_:CuSO_4_-5H_2_O = 10:1). The mixture was moistened and then treated with 5 mL of H_2_SO_4_ at 375 °C to eliminate boiling. A fixed volume of 50 mL of the resulting solution was analyzed using a flow injection analyzer. Total phosphorus (TP) determination: The alkali fusion—molybdenum antimony anti-colorimetric method was employed. Approximately 0.1500 g of air-dried soil sample was placed in a silver dry pan, a few drops of alcohol were added, and 2 g of NaOH was placed on top of the sample. The mixture was heated at 750 °C in a muffle furnace for 15 min, followed by cooling and dilution with sulfuric acid solution. A fixed volume of the filtered solution was analyzed using the molybdenum antimony anti-colorimetric assay with a spectrophotometer. Total potassium (TK) determination: The same pre-treatment as the determination of total phosphorus was conducted. A fixed volume of the resulting solution was analyzed using a flame photometer. Available phosphorus (AP) determination: About 5.00 g of air-dried sample was extracted with 50 mL of a solution of ammonium fluoride hydrochloric acid at (25 ± 1) °C. The mixture was shaken for 30 min, filtered, and a 10 mL aliquot of the filtrate was used for the molybdenum antimony anti-colorimetric assay with a spectrophotometer. Available potassium (AK) determination: About 5 g of the air-dried soil sample was extracted with 50 mL of a 1.0 mol/L ammonium acetate solution. The mixture was shaken for 30 min, filtered, and the resulting solution was analyzed using a flame photometer. pH determination: About 10 g of air-dried soil sample was mixed with 25 mL distilled water. After stirring for 1 to 2 min with a glass rod, the mixture was allowed to stand for 30 min. The pH of the resulting solution was measured using a pH meter [53].

### 4.4. Soil DNA Extraction, Sequencing, and Data Processing

Nucleic acids were extracted from between 0.2 and 0.5 g of soil samples using the OMEGA Soil DNA Kit (D5635-02) (Omega Bio-Tek, Norcross, GA, USA). For molecular size determination, the extracted DNA was electrophoresed on a 0.8% agarose gel, and its concentration was quantified using Nanodrop. The highly variable V3V4 region of the bacterial rRNA 16S gene, approximately 468 bp in length, was chosen for sequencing as part of the soil bacteria project [54]. PCR amplification was carried out using the 338F (5′-barcode + ACTCCTACGGGAGGCAGCA-3′) and 806R (5′-GGACTACHVGGGTWTCTAAT-3′) [55] specific primers targeting the 16S rRNA V3-V4 region of bacteria. In the case of the soil fungal project, the ITS1 fungal region has primers ITS5 (GGAAGTAAAAGTCGTAACAAGG) and ITS2 (GCTGCGTTCTTCATCGATGC) [56], with a length of about 280 bp, which were utilized for amplification and sequencing. Once components of the PCR reaction were set up, template DNA was pre-denatured at 98 °C for 5 min to ensure complete denaturation followed by the cycle of amplification. Quantification of PCR products was performed using a Microplate reader (BioTek, FLx800) with the Quant-iT PicoGreen dsDNA Assay Kit. For each sample, the quantified products were mixed based on the amount of data desired and used for library preparation using Illumina’s TruSeq Nano DNA LT Library Prep Kit. The microbiome data were analyzed using QIIME2 version 2019.4, following a modified and refined process based on the official tutorial (https://docs.qiime2.org/2019.4/tutorials/, accessed on 14 June 2023) [57]. High throughput sequencing was carried out on the Gene Cloud platform supplied by Shanghai Paisano Biotechnology Co. Alpha diversity indices including the observed species index, Shannon–Wiener index, Simpson’s index, Pielou’s evenness index, and Chao1 index were calculated using QIIME2 (2019.4) to evaluate the diversity of the microbiota within the samples.

### 4.5. Calculation of Soil Quality Index (SQI)

In general, the calculation of the soil quality index (SQI) consists of three steps [9,14]. The steps are as follows: (1) select a minimum data set (MDS) that best represents the soil function using principal component analysis (PCA), (2) score the selected indicators, and (3) compute SQI using the weighted index method. Seven soil chemical properties were analyzed (Table 2) and PCA was performed to identify the most appropriate indicators of soil quality. Principle components (PCs) with eigenvalues greater than 1.0 (explaining more than 10% of the total variance) were considered for the selection of indicators [58]. Pearson correlation analysis was employed to screen the indicators when multiple indicators were retained within each PC. If highly weighted indicators were not correlated (correlation coefficient less than 0.6), all indicators were included in the MDS. However, if there was a significant correlation, only the indicator with the highest weight value was retained in the MDS.

After selecting the MDS indicators, a nonlinear scoring function was applied to convert the soil indicators to a scale ranging from 0 to 1. The formula for the calculus is:

(1) $$S=a/{\left[1+\left(\frac{x}{x_{0}}\right)\right]}^{b}$$

where *S* is the score of the indicator, *a* is the maximal score (*a* = 1), *x* is the value of the soil indicator, *x*_0_ is the mean value of each indicator, and b is the slope value of the equation. For example, the slope value (*b*) is set to −2.5 and 2.5 to represent the “more is better” and “less is better” curves, respectively [59].

After all MDS indicators have been scored and weighted, the SQI is calculated according to the following formula [60]:

(2) $$SQI=\sum_{i=1}^{n}S_{i}\times W_{i}$$

where *S_i_*, *W_i_*, and *n* represent the score, weight, and number of indicators chosen, respectively.

### 4.6. Calculation of Productivity

To assess tree growth and productivity in this study, annual ring width increments were converted into basal area increments (BAIs), which are less influenced by age and tree size compared to annual ring width alone [61]. BAI was used as an index to quantify productivity. BAIs were calculated at the individual tree level using the following equation.

(3) $${BAI}_{i}=\pi \times \left(R_{n}^{2}-R_{n-T}^{2}\right)/T$$

where *BAI_i_* represents the average annual basal area increment (cm^2^/year) of the ith sampled tree within *T* years, *R_n_* and *R_n−T_* represent the radius at *n* years and *n − T* years, respectively, and *T* represents the study period.

To obtain the BAI at the stand level, the BAI of all sampled trees was weighted based on trees with the same diameter order using DBHc/DBHss. DBHc represents the square of the diameter of the subject tree, whereas DBHss is the average of the square of the diameter of all the trees in the stand at that order of diameter [23].

### 4.7. Statistical Analysis

A one-way analysis of variance (ANOVA) was used to analyze the effect of stand age on a variety of parameters including BAI (basal area increment), soil chemical properties, SQI, microbial community composition, and their diversity in tamarack stands. An honestly significant difference (HSD) Tukey’s test was used to determine significant differences between the means at a 5% level of significance. SPSS 26.0 software was used for statistical analysis, and data visualization was carried out using Origin software. In order to visualize similarities in bacterial and fungal community structure between different stand ages, multidimensional non-metric scaling (NMDS) based on the Bray–Curtis dissimilarity matrix was carried out using the ‘vegan’ package within the software package R. Similarity analysis (ANOSIM) was used to test whether the composition of the bacterial and fungal communities differed significantly between stand ages. We used the ‘vegan’ package in R to calculate the amount of variation explained (*R*^2^) and the significance (*p*) of the clustering scheme on the variance of the distance matrix, with 999 permutation tests [62]. Spearman correlation analysis was performed to evaluate the correlation between microbial α-diversity, community composition, and chemical properties of the soil. We conducted a redundancy analysis (RDA) to study the influence of soil chemical properties on the composition of microbial communities. Pearson’s correlation coefficient was used to investigate the relationship between the BAI and the independent variables. Before building the multiple regression model (Figure S1), the data were transformed to meet assumptions of normal distribution by logarithmic transformation of the dependent variable and standardization of the independent variables. Multiple linear regression models were developed using ordinary least squares (OLS) with a full subset regression selection model implemented through the ‘regsubsets’ function in the leaps package of the R software (Figure S2). Multicollinearity among variables was assessed using the ‘vif’ function in the car package of R, ensuring that all variance inflation factors were below 5, indicating no significant covariance among variables (Table S4). The relative importance of variables was evaluated using the ‘relaimpo’ package in R [63].

## 5. Conclusions

This study provides insights into the distribution patterns of microbial community composition, microbial diversity, and soil chemical properties across the age gradient in larch plantations, shedding light on the interconnections between these factors and enhancing our understanding of the relationship between microbial communities and stand productivity in this region. We observed a gradual increase in soil nutrient content with stand development, followed by a decline after 39 years. Therefore, it is crucial to implement enhanced management practices for stands older than 39 years to maintain soil quality. Acidobacteria were identified as nutrient-poor bacteria, while Proteobacteria were associated with a symbiotic lifestyle. However, in our study, Acidobacteria did not exhibit a nutrient-poor lifestyle due to the high soil nutrient levels. Ascomycota, characterized as a saprophytic fungus, which contributes to the decomposition of litters and SOM, and Basidiomycota, a mycorrhizal fungus specializing in lignin decomposition, which is not easily decomposed in litters, were both more abundant in old-growth stands (>80 years) due to the accumulation of large amounts of organic matter in these forests. The influence of stand age on bacterial diversity was found to be more significant compared to fungal diversity. Correlation analysis showed that microbial communities were closely related to soil chemical properties. Key soil quality indicators such as AK, SOM, TN, TP, and pH largely explained the changes in bacterial microbial communities, while TK, AK, SOM, TN, TP, and pH predominantly accounted for changes in fungal microbial communities. Fungal diversity exhibited a significant positive effect on forest productivity, while microbial diversity played an important role in determining forest productivity in larch plantations.

## Figures and Tables

**Table 1:** Soil chemical properties in larch stands with different ages.

Stand Age (Years)	14	25	31	39	>80
SOM (g kg^−1^)	33.54 ± 14.16 c	47.65 ± 14.92 bc	56.00 ± 11.47 ab	75.13 ± 11.94 a	67.73 ± 2.27 ab
TN (g kg^−1^)	2.81 ± 1.16 c	3.79 ± 0.98 bc	4.52 ± 0.90 ab	5.68 ± 0.46 a	4.27 ± 0.29 abc
TP (g kg^−1^)	0.50 ± 0.18 c	0.70 ± 0.16 abc	0.81 ± 0.19 ab	0.98 ± 0.09 a	0.66 ± 0.05 bc
TK (g kg^−1^)	17.32 ± 1.39	16.43 ± 1.40	17.35 ± 1.00	18.38 ± 2.72	16.37 ± 0.45 NS
AP (g kg^−1^)	9.79 ± 5.41 ab	8.34 ± 4.84 b	11.84 ± 5.15 ab	17.44 ± 6.87 a	8.86 ± 2.14 ab
AK (mg kg^−1^)	144.16 ± 46.67 ab	188.23 ± 25.05 ab	209.09 ± 54.72 a	212.63 ± 35.42 a	124.23 ± 21.43 b
PH	6.73 ± 0.45 a	6.39 ± 0.22 ab	6.32 ± 0.21 ab	6.07 ± 0.28 b	6.13 ± 0.24 b

Values are means ± standard deviation (*n* = 3); Different letters indicate significant differences (*p* < 0.05) among stand ages based on a one-way ANOVA followed by a Tukey test. Abbreviations: SOM, soil organic matter; total carbon; TN, total nitrogen; TP, total phosphorus; TK, total potassium; AP, available phosphorus; AK, available potassium. NS = not significant (*p* > 0.05).

**Table 2:** Basic stand information of the sampling plots.

Stand Age (years)	14	25	31	39	>80
Number of plots	8	8	12	4	8
Slope (°)	23 ± 5	26 ± 9	25 ± 5	18 ± 5	11 ± 2
Mean elevation (m)	1664 ± 21	1950 ± 111	1966 ± 191	2044 ± 34	2200 ± 97
Mean DBH (cm)	10.95 ± 0.98	13.48 ± 2.03	18.13 ± 2.31	24.71 ± 2.26	28.81 ± 2.50
Mean H (m)	8.54 ± 0.86	10.74 ± 1.33	13.01 ± 1.34	18.78 ± 1.15	28.77 ± 0.52
Mean stem density (tree·ha^−1^)	1763 ± 381	2144 ± 491	1187 ± 548	613 ± 224	822 ± 151

DBH: diameter at breast height; H: height of tree.

## Data Availability

Data are available upon request to the corresponding authors.

## Supplementary Materials

The following supporting information can be downloaded at: https://www.mdpi.com/article/10.3390/plants12162913/s1, Table S1: Results of principal components analysis of soil quality indicators in 0–10 cm layer of the larch plantation chronosequence; Table S2: Normalization equation of scoring curves; Table S3: Summary results (F-values and significance levels) for the relative abundance of soil bacteria, fungi from an analysis of variance (ANOVA); Table S4: The results of variance inflation factors for larch forests; Table S5: Summary of the best ordinary least squares (OLS) multiple regression model for the effects of Soil nutrient, stand age, stand density, bacterial and fungal microbial diversity on forest productivity; Figure S1: Pearson correlation coefficients (r) among measured variables in larch forests. Note: BAI, basal area increment; B, Bacteria; F, Fungal; SQI, soil quality index. All data have been logarithmically converted. * *p* < 0.05; Figure S2: 16 candidate models were screened using full subset regression.
