# Supplementary material for: Soil Microbial Community, Soil Quality, and Productivity along a Chronosequence of Larix principis-rupprechtii Forests
Source: Plants (Basel). 2023 Aug 10;12(16):2913. doi: 10.3390/plants12162913 (PMC10458017; doi:10.3390/plants12162913)
Supplement: Supplementary file 1 [file plants-12-02913-s001.zip › plants-2479539-supplementary.pdf]

Supplementary:

Table S1. Results of principal components analysis of soil quality indicators in 0-10 cm layer of the larch plantation chronosequence.

Principal components	PC-1	PC-2
Eigenvalues	4.09	1.18
Variance (%)	58.41	16.82
Cumulative (%)	58.41	75.22
Weighting value	0.58	0.17
SOM	0.91	-0.07
TN	**<u>0.96</u>**	0.04
TP	0.89	0.14
TK	-0.02	**<u>0.92</u>**
AP	0.59	0.22
AK	0.79	0.19
PH	-0.75	0.47

Notes: Bold values indicate as highly weighted loading factors and underlined values were the factors retained in MDS.

Table S2. Normalization equation of scoring curves.

Parameter	Average (x_0)	Curve type	Slope (b)	Normalization equation	Weighting value (*W*)
TN	3.86	More is better	-2.5	$S = 1/(1+(x/3.86)^{-2.5})$	0.58
TK	17.56	More is better	-2.5	$S = 1/(1+(x/17.56)^{-2.5})$	0.17

Table S3. Summary results (F-values and significance levels) for the relative abundance of soil bacteria, fungi from an analysis of variance (ANOVA). * $p < 0.05$

Bacteria phylum		Fungi phylum	
Proteobacteria*	2.82	Ascomycota*	3.86
Acidobacteria*	4.92	Basidiomycota	1.10
Actinobacteria*	18.60	Mortierellomycota*	5.55
Chloroflexi*	29.64	Mucoromycota	0.62
Gemmatimonadetes*	8.70	Rozellomycota	2.13

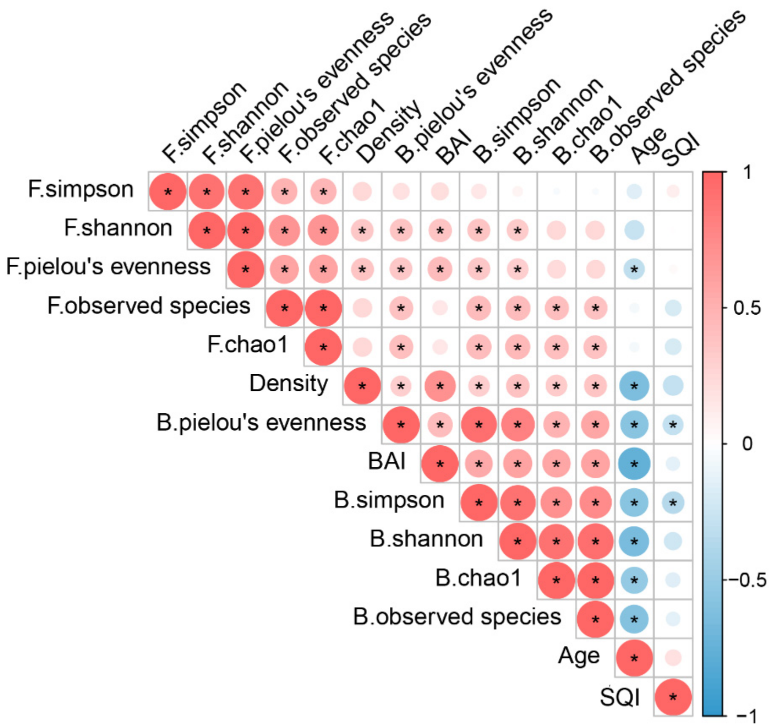

Figure S1. Pearson correlation coefficients (r) among measured variables in larch forests. Note: BAI, basal area increment; B, Bacteria; F, Fungal; SQI, soil quality index. All data have been logarithmically converted. * $p < 0.05$.

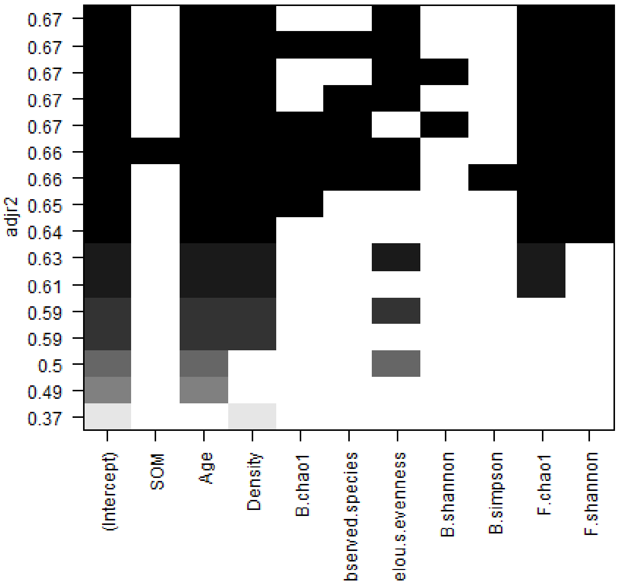

Figure S2. 16 candidate models were screened using full subset regression.

Table S4. The results of variance inflation factors for larch forests.

Variable	VIF value
SQI	2.44
Age	2.29
Density	1.52
Bacterial pielou's evenness	1.44
Fungal chao1	2.08
Fungal shannon	2.03
CWM_LCC	1.56
CWM_LKC	1.08

Table S5. Summary of the best ordinary least squares (OLS) multiple regression model for the effects of Soil nutrient, stand age, stand density, bacterial and fungal microbial diversity on forest productivity.

Variable	Estimate	SE	*t*-value	*p*-value
Intercept	2.694	0.491	5.491	<0.001***
SQI	0.035	0.017	2.115	0.042*
Age	-0.071	0.018	-3.878	<0.001***
Density	0.062	0.016	3.858	<0.001***
Bacterial pielou's evenness	-0.072	0.027	-2.679	0.011*
Fungal chao1	-0.695	0.198	-3.502	0.001**
Fungal shannon	0.329	0.161	2.038	0.049*

*** $p < 0.001$; ** $p < 0.01$; * $p < 0.05$; Ns, not significant.
